# Bovine Genomics by James E. Womack

**DOI:** 10.3389/fgene.2012.00275

**Published:** 2012-11-27

**Authors:** Hasan Khatib

**Affiliations:** Department of Animal Sciences, University of Wisconsin-MadisonMadison, WI, USA

**A book review on**

**Bovine Genomics**

Edited by James E. Womack, Oxford, UK: Wiley-Blackwell, 2012, 271 pages. ISBN: 978-0813821221

*Bovine Genomics* consists of 15 chapters edited by James Womack, with contributions from a diverse, unique, and distinguished group of scientists. James Womack is a member of the National Academy of Sciences USA and a recipient of several prestigious international awards including the CIBA and Wolf prizes for his contributions to the advancement of animal health and agriculture. Womack is a visionary scientist whose work has enabled many of the current bovine research achievements through his initial studies on the organization of the bovine genome and his intensive research of comparative genomic approaches. The research vision of Womack is sincerely reflected in the contents of this book.

*Bovine Genomics* is not only a collection of book chapters covering different topics of the bovine research; the contents and contributors of this book are a reflection of the extent of the involvement of Womack in the international effort to advance cattle research. A journey into *Bovine Genomics* starts thousands of years ago with archeology and the domestication of cattle to current genome association studies, genomic selection, bovine epigenetics, and the identification of quantitative trait nucleotides affecting phenotypic variation in cattle. For example, Frank Nicholas (Chapter 2) discusses the molecular basis of Mendelian traits in cattle with specific trait and gene examples, followed by Sheila Schmutz (Chapter 3) describing recent research on the genetics of coat color in cattle including specific genes and mutations. The cytogenetic analysis of the bovine X and Y chromosomes are thoroughly described in chapter 7, including mapping of the Y chromosome, sex chromosome abnormalities, and sex chromosome QTL. Goddard and Hayes (Chapter 13) extensively discuss the different aspects of genome-wide association studies and linkage disequilibrium in cattle including experimental design and statistical analysis of these studies, and they provide a summary of the results of over 30 studies reported in cattle. In Chapter 14, Taylor and colleagues provide a thorough review of genomic selection in beef cattle. The authors review genomic selection theory, methods of genomic selection, benefits and drawbacks, and discuss the future of genomic selection in beef cattle.

Given the wide range of topics covered in this book, it is difficult to exclude a group of people who would not benefit from it. The book should be of interest to undergraduate students, graduate students, and professionals who are working in the field of bovine genomics. In addition, researchers of other livestock species can benefit from the research strategies presented in this book such as studying the molecular mechanisms of single traits, QTL mapping, genome-wide association analysis, and sequencing of the bovine genome.

It is worth noting that information delivery is not the only important aspect of this book. Readers, specifically graduate students and early career scientists, can gain perspective and acquire insight into the accumulating scientific life experience of many of the book's contributors. Without exception, the contributors to this book are well-established, leading researchers in the field of cattle genetics and genomics. In this review I will only mention a few of them. In chapter 4 “From Quantitative Genetics to Quantitative Genomics: A Personal Odyssey” by Morris Soller, you can trace “the story of my life” of a brilliant scientist who read T. H. Morgan's book *The Theory of the Gene* at age 12. Soller writes a unique documentation of his own scientific career along with a detailed description of the development of the theory and applications of marker assisted selection and animal breeding. Soller is a true scientist who has proposed novel research ideas for over 40 years, which have contributed unprecedentedly to the whole scientific community. His early ideas that genetic markers can be used to improve economically important traits have paved the way to the new discoveries of causative genes affecting production traits.

A leader and distinguished scientist who successfully developed novel approaches of identification of causative genes underlying phenotypic variation in livestock is Michel Georges. In Chapter 15, Georges presents an extensive discussion on QTL mapping, positional cloning and the candidate gene approach, the impact of high-density SNP genotyping on the analysis of monogenic and complex traits, distinguishing correlation from causation, and the impact of next-generation sequencing on the analysis of complex traits. The chapter contents of Georges reflect aspects of his successful career as a leader in this field.

In summary, what makes this book special is the unique combination of a distinguished editor, contributors who are leading researchers in bovine genomics and the variety of topics covered in this book. The release of the bovine genome sequence has had a remarkable influence on the quantity and quality of genetics research in cattle. Thus, future revisions can include additional topics such as genetics of disease resistance, epigenetics and production traits, and the use of cattle as an animal model in biomedical research. The chapters of this book fit together to tell a cohesive story of the past, present, and future of bovine genome research.

